# Supplementary figures and images for: Spatio-temporal dynamics of early somite segmentation in the chicken embryo
Source: PLoS One. 2024 Apr 18;19(4):e0297853. doi: 10.1371/journal.pone.0297853 (PMC11025740; doi:10.1371/journal.pone.0297853)

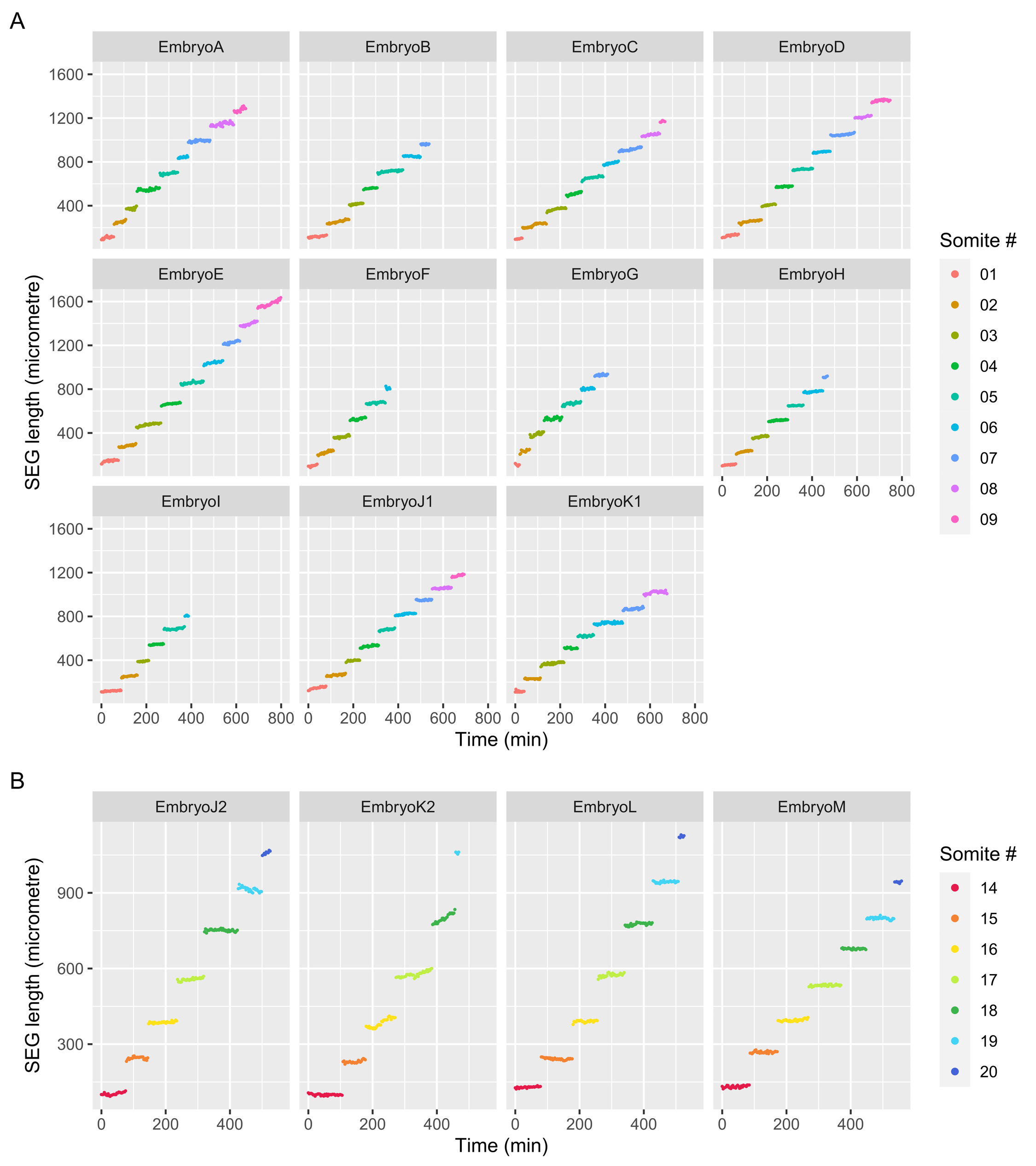

Supplement: S1 Fig — Length of the segmented region (SEG) of chicken embryos, from 1 to 9 somites (A) and 14 to 20 somites (B), over time. Embryos J and K were cultured continuously from HH7 (1 somite-stage) until HH13+ (20 somite-stage). J1 and K1 correspond to measurements from somites 1–10; J2 and K2 correspond to measurements from somites 14–20. (TIF) [file pone.0297853.s001.tif]

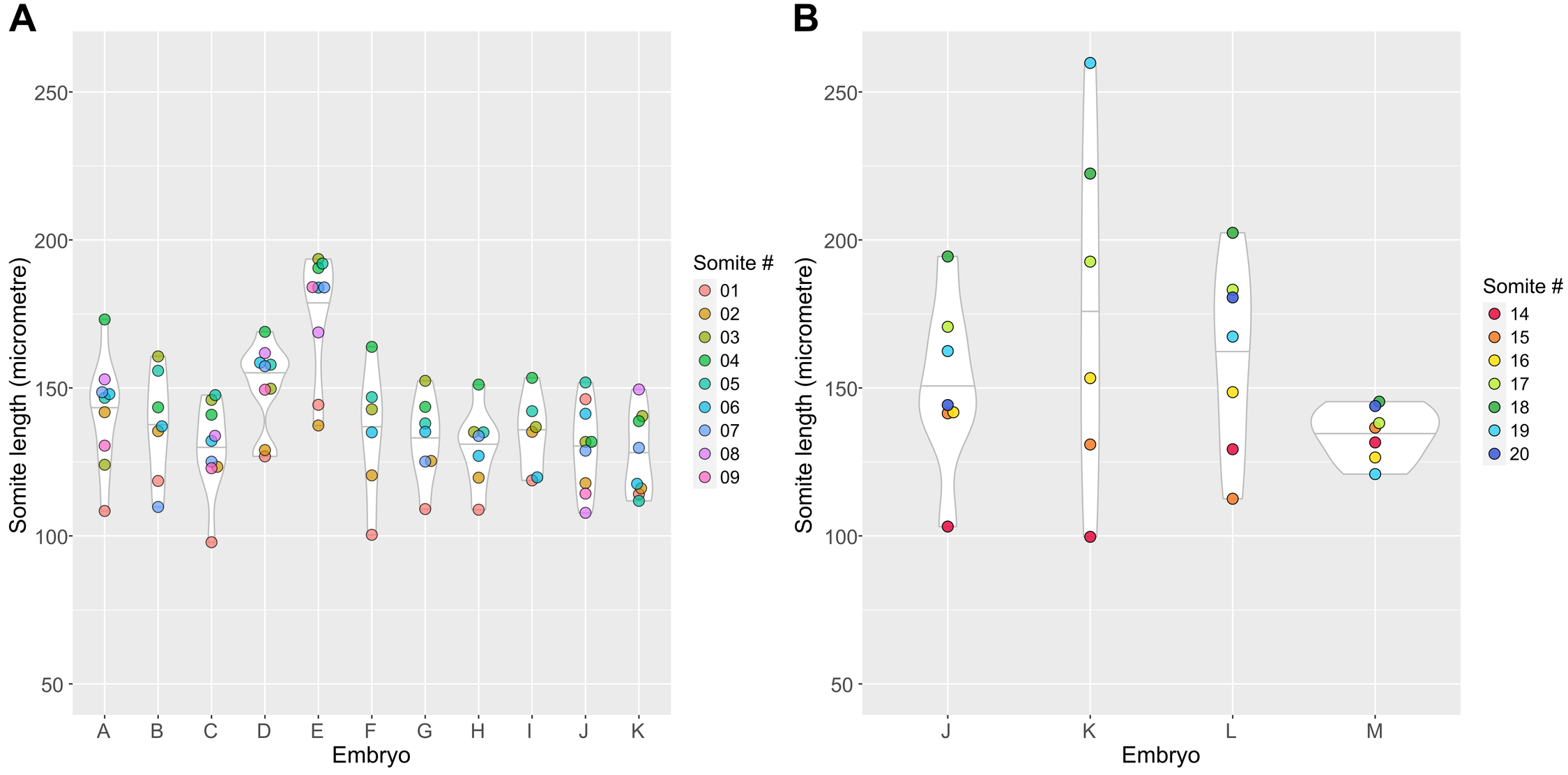

Supplement: S2 Fig — Violin-plot distribution of the observed lengths of somites 1–9 (A) and 14–20 (B) in each of the embryos analysed. (TIF) [file pone.0297853.s002.tif]

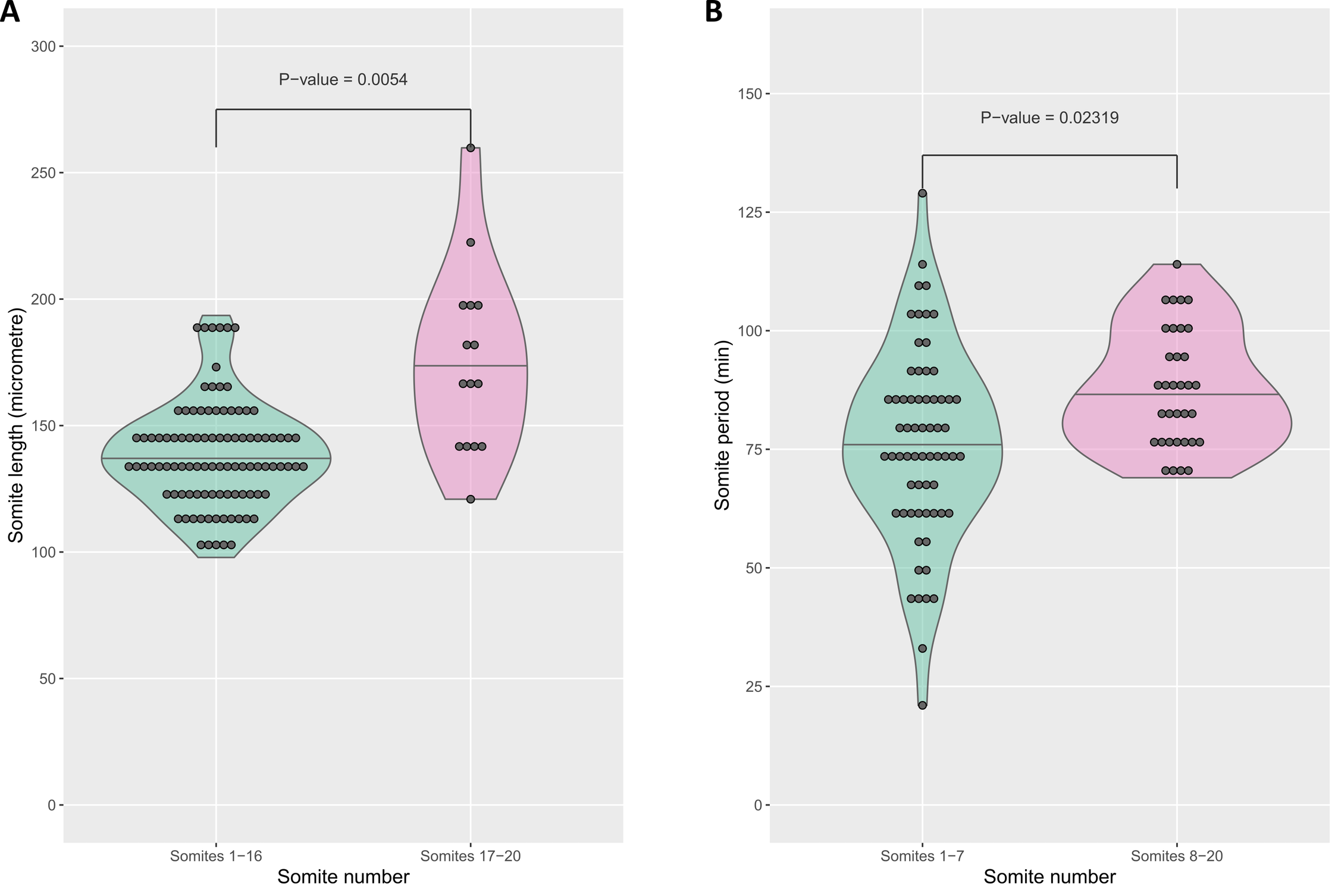

Supplement: S3 Fig — Brown-Forsythe test for homogeneity of variance of somite length (A) and formation period (B). There is a statistically significant difference between the variance of the (A) measured lengths of somites 1–16 and 17–20 (F value = 8.0527; Pr(>F) = 0.005403), and of the (B) formation period of somites 1–7 versus 8–20 (F value = 5.3231; Pr(>F) = 0.02319). (TIF) [file pone.0297853.s003.tif]

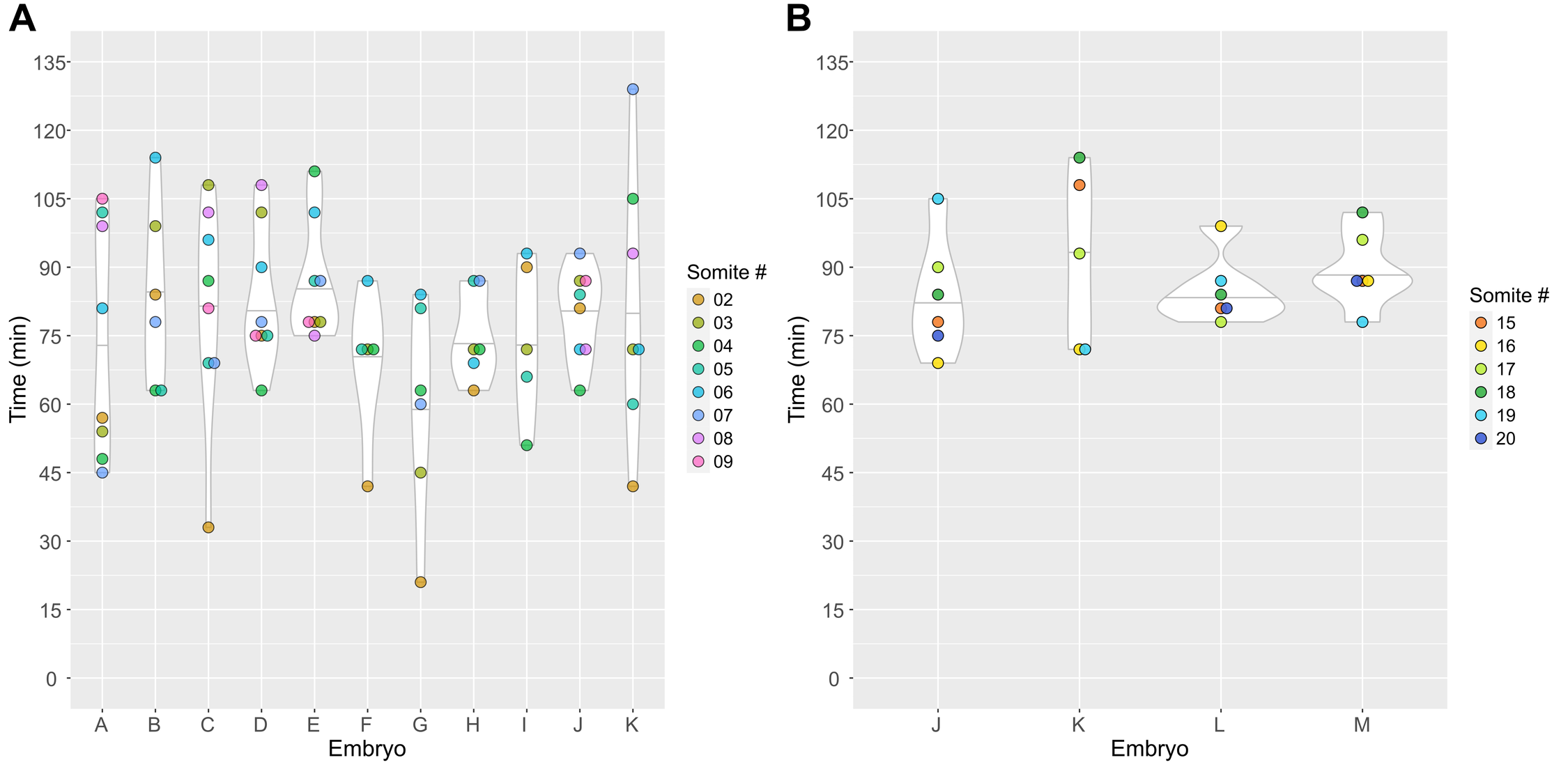

Supplement: S4 Fig — Violin-plot distribution of the observed periods of somites 2–9 (A) and 15–20 (B) in each of the embryos analysed. (TIF) [file pone.0297853.s004.tif]

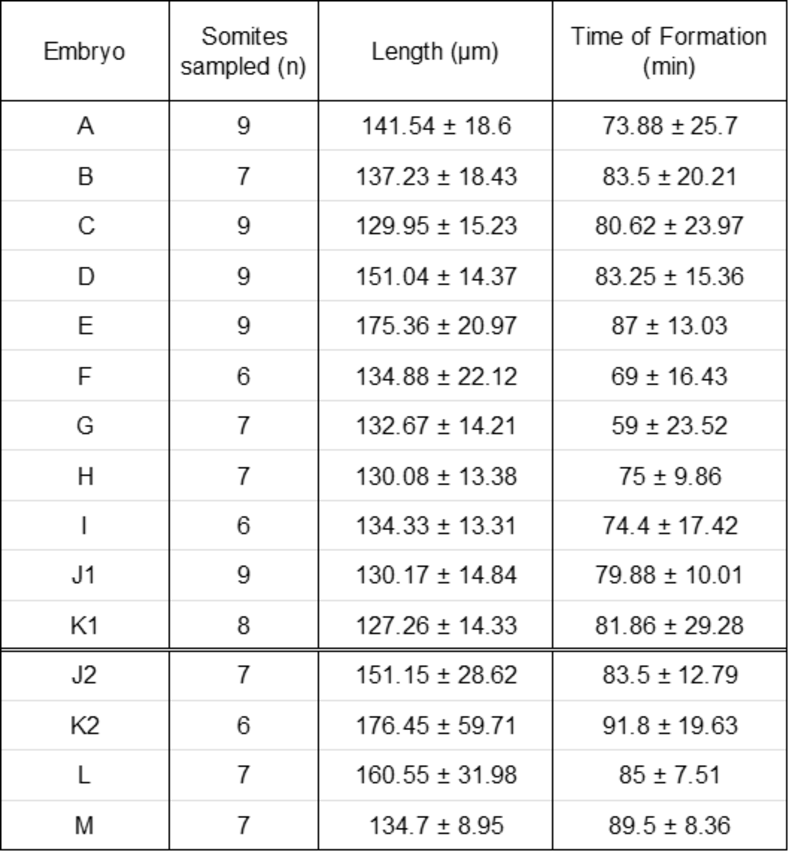

Supplement: S1 Table — Mean ± standard deviation is shown. Embryos J and K were cultured continuously from HH7 (1 somite-stage) until HH13+ (20 somite-stage). J1 and K1 correspond to measurements from somites 1–10; J2 and K2 correspond to measurements from somites 14–20. (TIF) [file pone.0297853.s005.tif]

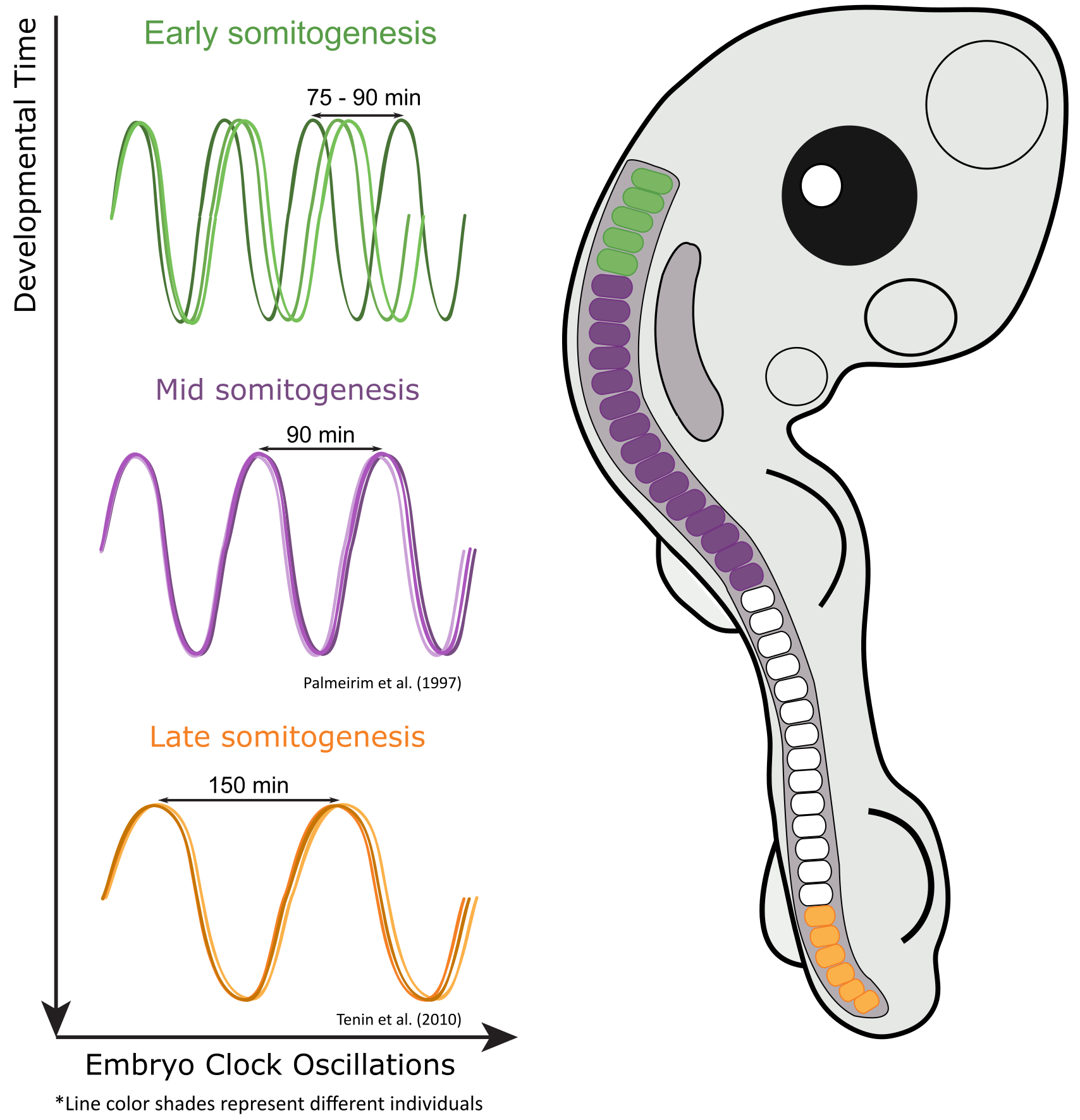

Supplement: S1 Graphical abstract — (TIF) [file pone.0297853.s006.tif]
